# Ultra-high-field 7-T MRI in multiple sclerosis and other demyelinating diseases: from pathology to clinical practice

**DOI:** 10.1186/s41747-020-00186-x

**Published:** 2020-10-22

**Authors:** Nicolo’ Bruschi, Giacomo Boffa, Matilde Inglese

**Affiliations:** 1 Department of Neuroscience, Rehabilitation, Ophthalmology, Genetics, Maternal and Child Health (DINOGMI), University of Genoa, Genoa, Italy; 2 Ospedale Policlinico San Martino, IRCCS, Largo Daneo 3, 16100 Genoa, Italy

**Keywords:** Ultra-high-field magnetic resonance imaging, Demyelinating diseases, Multiple sclerosis, Neuromyelitis optica spectrum disorders, Systemic autoimmune diseases

## Abstract

Magnetic resonance imaging (MRI) is essential for the early diagnosis of multiple sclerosis (MS), for investigating the disease pathophysiology, and for discriminating MS from other neurological diseases. Ultra-high-field strength (7-T) MRI provides a new tool for studying MS and other demyelinating diseases both in research and in clinical settings. We present an overview of 7-T MRI application in MS focusing on increased sensitivity and specificity for lesion detection and characterisation in the brain and spinal cord, central vein sign identification, and leptomeningeal enhancement detection. We also discuss the role of 7-T MRI in improving our understanding of MS pathophysiology with the aid of metabolic imaging. In addition, we present 7-T MRI applications in other demyelinating diseases. 7-T MRI allows better detection of the anatomical, pathological, and functional features of MS, thus improving our understanding of MS pathology *in vivo*. 7-T MRI also represents a potential tool for earlier and more accurate diagnosis.

## Key points

- 7-T magnetic resonance imaging (MRI) allows better multiple sclerosis (MS) lesion detection and staging in the brain and spinal cord.
- 7-T MRI allows better central vein sign identification and leptomeningeal inflammation detection and characterisation in MS.
- 7-T MRI improves *in vivo* imaging of cortical lesion in MS.
- 7-T MRI iron imaging helps differentiating MS from its mimickers including neuromyelitis optica spectrum disorders, systemic lupus erythematosus, and Susac syndrome.
- ^23^Na 7-T MRI may help in understanding MS pathology.

## Background

Multiple sclerosis (MS), the most common demyelinating disease of the central nervous system (CNS), is a neuroinflammatory/neurodegenerative disease that affects more than two million people worldwide [1]. Environmental factors, a favourable genetic background, and its connections play a key role in MS onset pathogenesis. The first pathological event is represented by the transmigration of myelin-autoreactive T lymphocytes across the blood-brain barrier (BBB) with the induction of an acute autoimmune reaction against CNS myelin. From a clinical point of view, MS is characterised by unpredictable clinical and radiological relapses followed by variable recovery (relapsing-remitting MS), but over time, relapses tend to occur less frequently and are replaced by a gradual neurological worsening (secondary progressive MS). A minority of patients experience a subtle progressive worsening of disability by the onset of the disease (primary progressive MS) [2]. Similarly to MS, neuromyelitis optica spectrum disorders (NMOSD) represent a spectrum of inflammatory, demyelinating syndromes of the CNS that are characterised by severe attacks of recurrent optic neuritis and myelitis [3]. NMOSD affects primarily non-white populations, in whom MS is rare and it is an antibody-mediated disorder with the main target being the astrocytes with the associated channels and proteins such as aquaporin (AQP). Although a secondary progressive phase is rare in NMOSD, recovery from relapses is usually incomplete, and most patients experience an early accrual of disability due to frequent and severe relapses. Magnetic resonance imaging (MRI) plays an essential role in the management of demyelinating disease. Indeed, MRI facilitates early diagnosis, helps with differential diagnoses, and provides an easy and reliable tool to monitor treatment response in clinical practice and experimental trials. The use of ultra-high-field 7-T MRI is gaining growing popularity in investigating disease mechanisms in patients with MS and other demyelinating diseases. The main advantages of 7-T MRI are the increased signal-to-noise ratio and contrast-to-noise ratio that allow a better spatial resolution and detection of anatomical and pathological features. In this review, we will first briefly illustrate the state of the art in MS and NMOSD diagnosis with standard MRI using 1.5-T and 3-T magnets. Then, we will describe the current development of 7-T MRI techniques regarding MS white (WM) and grey matter (GM) pathology in the brain and spinal cord, detection of the central vein sign (CVS), and leptomeningeal enhancement (LME) and the application of multinuclear imaging. In addition, we will give an overview of the use of 7-T MRI for other neurological autoimmune diseases, with a special focus on NMOSD.

## MS diagnosis

According to the current MS diagnostic criteria (2017 McDonald criteria), it is possible to diagnose MS in a patient who presents with symptoms consistent with a CNS inflammatory demyelinating disease or a typical clinically isolated syndrome coupled with the demonstration of disease dissemination in space and time and with the exclusion of alternative diagnoses [4]. The *clinically isolated syndrome* is characterised by symptoms involving the optic nerve, brainstem, cerebellum, spinal cord, or cerebral hemispheres and can be unifocal or multifocal. Radiological *dissemination in space* is defined as one or more T2 hyperintense lesions within at least two of five anatomical locations characteristic for MS: periventricular, juxtacortical, cortical, infratentorial, and the spinal cord. Radiological *dissemination in time* is demonstrated by the presence of (i) gadolinium-enhancing and nonenhancing lesions on the same MRI examination or (ii) new T2 hyperintense and/or gadolinium-enhancing lesions on follow-up MRI compared to baseline, regardless of the time interval between the two scans. Dissemination in time can be also demonstrated by the presence of oligoclonal bands in the cerebral spinal fluid (CSF). In order to facilitate the application of these criteria in clinical settings, guidelines for the standardisation of MRI protocols utilised in MS diagnosis and follow-up have been published [5]. More specifically, the protocol should preferably be performed on a 3-T scanner, and it should be completed in 25–30 min with maximum slice thickness of 3 mm and an in-plane spatial resolution of 1 × 1 mm^2^ (voxel size 3 × 1 × 1 mm^3^). Details of specific sequences for baseline and follow-up examinations are resumed in Table 1 [5].

**Table 1:** Standardised protocol for brain MRI

Brain MRI is important for achieving a prompt, accurate diagnosis of MS. Here we present a standardised protocol for the evaluation of patients with suspected or clinically definite MS; however, the precise sequences and timing of follow-up scans must be determined on a case-by-case basis	
**Baseline evaluation**	
Mandatory sequences	
▪ Axial proton-density and/or T2-FLAIR/T2-weighted	
▪ Sagittal 2D or 3D T2-FLAIR	
▪ 2D or 3D contrast-enhanced T1-weighted	
Optional sequences	
▪ Unenhanced 2D or high-resolution isotropic 3D T1-weighted	
▪ 2D and/or 3D dual inversion recovery	
▪ Axial diffusion-weighted imaging	
**Follow-up examinations**	
Mandatory sequences	
▪ Axial proton-density and/or T2-Flair/T2-weighted highly recommended	
▪ 2D or 3D contrast-enhanced T1-weighted	
Optional sequences	
▪ Unenhanced 2D or high-resolution isotropic 3D T1-weighted	
▪ 2D and/or 3D dual inversion recovery	
▪ Axial diffusion-weighted imaging	

*Abbreviation*: *FLAIR* Fluid-attenuated inversion recovery, *MS* Multiple sclerosis

Although the use of 7-T MRI is not yet included in standard protocols for MS, the increased sensitivity in lesion detection and the better characterisation of lesion pathology at high- and ultra-high-field MRI could lead to higher specificity, thus enhancing differentiation of MS from other disorders.

## NMOSD diagnosis

Neuromyelitis optica diagnosis is based on the 2015 International Panel for Neuromyelitis Optica Diagnosis [6]. The criteria allow NMOSD diagnosis with the occurrence of at least one of the six core clinical characteristics of the disease, altogether with the detection of serum AQP4-IgG. The core clinical characteristics include involvement of the optic nerve, the spinal cord, the area postrema of the dorsal medulla, the brainstem, the diencephalon, and the cerebrum. Typical clinical presentations are bilateral optic neuritis, transverse myelitis, and area postrema clinical syndrome. In those patients where serum AQP4-IgG cannot be demonstrated, NMOSD diagnosis is allowed following two or more than two different core clinical characteristics. At least one of the two must be an optic neuritis, a transverse myelitis with a longitudinally extensive lesion seen on MRI, or an area postrema syndrome with an associated medullary lesion on MRI. Nevertheless, the differentiation between NMOSD and MS remains challenging, in view of overlapping clinical manifestations and findings on conventional neuroimaging. Indeed, nonspecific cerebral white matter (WM) lesions are present in nearly 70% of patients with longstanding NMOSD [7]. Differentiating MS from non-MS is feasible even with 3-T scanners using central vein sign-based criteria. Current evidence provides sufficient differentiation with a threshold of 35% of lesions with central vein sign (sensitivity 68.1%, specificity 82.9%) or more than three lesions with central vein sign (sensitivity 61.9%, specificity 89%) [8]. Although there are no comparative studies between 3-T and 7-T MRI for neuromyelitis optica diagnosis, due to its high signal-to-noise ratio, increased spatial resolution, and markedly improved venous and iron contrast within lesions, 7-T MRI has the potential to improve the detection and the morphologic characterisation of MS *versus* NMOSD lesions.

## MS lesion analysis

### Detection and staging

MRI is by far the most suitable instrument for *in vivo* evaluation of MS pathology, because of its high sensitivity and the possibility of longitudinal assessments. Multimodal imaging at 7-T has yielded biologically relevant insights into lesion structure and BBB dynamics, allowing better lesion detection and staging. Animal studies [9] have previously demonstrated that BBB permeability increases over the 4 weeks prior to lesion appearance and that these early changes in vascular permeability are associated with perivascular inflammatory cuffing and parenchymal microglial activation. At 7 T, focal alterations of MRI signal on T2-weighted fluid-attenuation inversion recovery (FLAIR) and T2*-weighted images have been found to precede the formation of discrete MS lesions in humans [10]. Specifically, the use of dynamic contrast-enhanced MRI, in which T1-weighted images are acquired before, during, and after injection of gadolinium-based contrast agent, has allowed a better characterisation of the spatiotemporal dynamics of BBB opening in active MS lesions. It was possible to demonstrate that, before the overt onset of the radiologically defined demyelinated lesion, the “inflamed” central vein within the future lesion enhances in isolation, perhaps owing to higher BBB permeability [11]. In this work, ten repetitions of a three-dimensional T1-weighted dynamic contrast-enhanced sequence (time resolution 32 s) were acquired during intravenous injection of 0.1 mmol/kg of gadobutrol. This sequence was further repeated at variable time points over 25 min after contrast administration [11]. When early active demyelinating lesions appear, gadolinium leaks initially from the central vein and flows outward, supporting the centrality of perivenular pathogenic events at lesion onset. After a few weeks from lesion formation, most lesions exhibit a shift in the pattern of gadolinium enhancement, with an expanding inflammatory edge which enhances first, with subsequent centripetally gadolinium flow. The significance of centripetal enhancement might reflect the wound-healing inflammatory reaction of the CNS parenchyma. Using T2*-weighted gradient echo sequences and susceptibility-weighted imaging at 7 T, a persistent hypointense rim is often seen in a subset of WM lesions [8, 12, 13]. Radiological-histopathological correlation studies have demonstrated that this hypointense rim reflects the presence of iron within phagocytic macrophages and/or microglia at the border of such MS lesions, providing a histologic validation for the radiological finding [12, 14]. A recent 3-T MRI and positron emission tomography (PET) study confirmed that rim lesions show an uptake of PET tracer (11C-PK11195) sensitive to the presence of activated microglia/macrophages [15]. It has been thus proposed that lesions with a hypointense rim represent the radiological proxy to the “chronic active lesions” described by pathologists. Indeed, after the initial phase of inflammatory demyelination, lesions can show different pathological and repair outcomes, namely “chronic active/slowly expanding/smouldering,” “chronic inactive,” and, when repair is successful, “remyelinated.” Chronic active lesions prevail in patients with higher disability [16] and are thus a potentially useful biomarker for patient stratification and monitoring. A recent study by Absinta et al. [17] confirmed *in vivo* that chronic active lesions are associated with more aggressive disease, exert ongoing tissue damage, and occur even in individuals treated with effective disease-modifying therapies. These results support using paramagnetic rim lesion development, or the resolution of such lesions, as outcome measures in MRI-based clinical trials. The optimal MRI sequence for the detection of chronic active lesions is still unknown. Evidence suggests that susceptibility-weighted imaging (SWI) and phase imaging are strongly affected by the susceptibility of structures usually surrounding MS lesions, such as the ventricles or iron-rich nuclei, and are dependent on lesion orientation and geometry because of dipole field changes [18]. Quantitative susceptibility mapping (QSM), on the other hand, is not susceptible to orientation and geometry effects, thus reducing the false positive detection of rim structure around lesions but is difficult to implement in routine MRI examination. The combined use of QSM and relaxation rate (R2*) is of particular interest because of the different effects myelin and iron have on tissue R2* and susceptibility: increased myelin content increases R2* but decreases susceptibility due to its diamagnetic nature, while increased iron content leads to increases in both R2* and susceptibility. This combined approach enables to disentangle myelin loss from iron loss/deposition within a single lesion, with some lesions being characterised by moderate/major demyelination without iron loss/deposition (hypo on R2* and hyper on QSM) and lesion with minor myelin loss and moderate/major iron deposition (hyper on R2* and on QSM) [18].

### The central vein sign

T2*-weighted gradient echo sequence and susceptibility-weighted imaging were the first sequences used on 7-T MRI to investigate the relationship between WM lesions and small parenchymal blood vessels in MS [11, 19–22]. The existence of a central vein within MS lesions has been known for more than a century [23], and the proximity between an MS lesion and a blood vessel has been demonstrated with T2-weighted sequence at 1.5 T with subsequent venography [24]. However, thanks to the application of T2*-weighted sequences at 7 T, it was possible to visualise both structures simultaneously. According to histologic reports, a central vessel can be detected in most of MS lesions [19]. In general, periventricular lesions tend to exhibit more frequently a central vessel than lesions in peripheral WM. Whether this finding depends on different mechanisms of lesion development in the periventricular zone or to the general smaller venule size in peripheral WM is not known yet. The first 7-T MRI studies raised questions about the clinical implication of this central vein sign (CVS) for an early MS diagnosis and for the exclusion of several MS mimickers. Indeed, although temporally distinct focal T2 lesions are the cornerstone of MRI-based MS diagnosis, these findings are not specific to MS, occurring in several other neurological disorders. The finding that a central vessel could be identified in 87% of WM lesions in MS patients and in only 8% of the WM lesions of healthy volunteers [19] prompted further investigation. Tallantyre et al. [20] demonstrated that 7-T T2*-weighted MRI distinguished patients with clinically definite MS (more than 40% of lesions presented the CVS) from those without MS (less than 40% of lesions presented CVS) with a 100% specificity. Of note, perivenous lesion appearance was more predictive of MS (odds ratio = 14, *p* < 0.001) than lesion location within the anatomical regions characteristic for MS according to the McDonald criteria. Although the CVS has been detected also at 3-T MRI, 7-T magnets allow better visualisation of the central vein within discrete lesions, providing a slightly higher CVS detection rate [19]. Several works using both 3-T and 7-T MRI aimed at evaluating the presence of central veins inside WM lesions associated with various neurological diseases, including NMOSD, systemic autoimmune diseases, cerebral small vessel disease, Susac syndrome, and migraine, supporting the concept that the CVS is highly specific for MS and may strongly contribute to MS differential diagnosis [25, 26]. The North American Imaging in MS (NAIMS) Cooperative has recently provided a consensus document which illustrates guidelines for CVS assessment [25]. Validation of the NAIMS criteria has been recently performed in a real-world setting, showing that nonconfluent lesions greater than 3 mm with at least one central vein were the most sensitive and specific differentiators between patients with MS and control subjects [27]. Recently, an international, multicentre cross-sectional 3-T MRI study within the MAGNIMS Study Group [8] was conducted with the aim to investigate the sensitivity and specificity of various CVS-based criteria in differentiating MS from MS mimickers. CVS showed a high specificity in differentiating MS from non-MS diseases either by applying a three-lesion-based threshold or a 35% CVS proportion threshold.

CVS can be assessed by T2*-weighted sequences or by a combination of T2*-weighted or SWI and FLAIR. It has been suggested that the sensitivity and specificity in detecting veins were highest on optimised T2*-weighted images and lower when a combined SWI and FLAIR approach was applied [8]. This is probably due to the abovementioned effects on susceptibility of lesion orientation and geometry. Prospective studies evaluating the detection rate of the CVS using different MRI sequences are warranted.

### Cortical lesions and cortical pathology

**Fig. 1:**
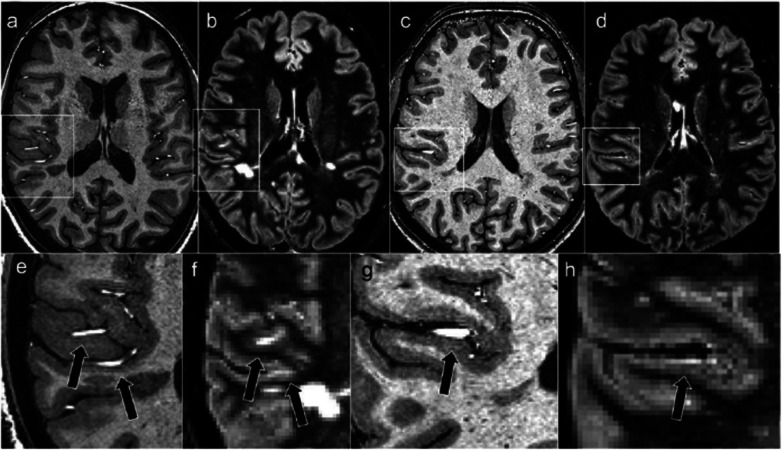
Cortical lesions and artefacts. Two hyperintensities misinterpreted as cortical lesions on a 3-T double inversion recovery images (**b**–**f**), are *de facto* extracranial vessels, as demonstrated on 7-T magnetisation-prepared rapid gradient echo images (**a**–**c**). Magnified versions of the images are also shown (**e**–**h**). Published with permission from *J Magn Reson Imaging* [41]

Due to an increased spatial resolution and better tissue contrast, 7-T MRI has allowed a more accurate detection and characterisation of cortical GM lesions [28–33]. Cortical lesions are very common at histopathological examination, especially in the progressive forms of MS [34]. Cortical lesions can be used to demonstrate dissemination in space accordingly to the 2017 McDonald criteria [4] and have not been reported so far in other conditions that might mimic MS, including NMOSD [35]. Moreover, cortical lesions are associated with disability progression [36] and cognitive impairment and could thus represent an important biomarker in the evaluation of progression of disease burden in MS. Focal cortical lesions are poorly visualised using conventional MRI sequences at 1.5 T [37] and 3 T, because of their small size, lower amount of inflammation than WM lesions, and partial volume effects with CSF. Since the first *ex vivo* and *in vivo* applications, it was clear that 7-T MRI improves not only detection of cortical lesions compared to 3-T MRI [29, 30, 38], but it also improves lesion localisation and classification [28, 30]. A recent study by Treaba et al. [32] found that cortical lesions predominantly develop intracortically and within sulci, supporting an inflammatory cerebrospinal fluid-mediated lesion pathogenesis of those lesions, as already suggested by MRI-pathological correlation studies [39, 40] (Fig. 1). Improved lesion localisation and classification is of paramount importance, given the fact that the different cortical lesion subtypes might have differing pathogenesis and clinical relevance. In the study by Harrison et al. [31], subpial lesion volume correlated with cortical GM atrophy but not with WM lesion volume, while leukocortical lesions strongly correlated with WM lesions without a significant correlation with cortical GM atrophy. Leukocortical lesions, but not subpial ones, were found to be strongly correlated with cognitive impaired, probably because of the involvement of subcortical U-fibres [31]. Cortical lesions, especially leukocortical ones, could have indeed an impact on wide-range WM bundles, as highlighted by a recent study [42] which has evaluated the microstructural tissue changes that accompany early cortical lesions in MS and found that cortical demyelination was associated with worse global connectivity metrics in WM. Most 7-T MRI studies used several sequences to detect and characterise cortical lesions, but results are still controversial and each sequence carries its own advantages and limitations. One study evaluated the agreement between 3-T FLAIR, 3-T double inversion recovery, and 7-T magnetisation-prepared rapid gradient echo sequences in MS patients and healthy controls [41]. This study showed that the agreement for leukocortical cortical lesion detection was good between the three images, while the agreement for intracortical detection and classification was less satisfactory, with up to 25% of lesions which could only be visualised on an individual MRI sequence. Each sequence gave a complementary contribution to lesion detection, with 7-T magnetisation-prepared rapid gradient echo (MPRAGE) facilitating the localisation of subpial cortical lesions. Moreover, since the lesion hypointensity visible on 7-T MPRAGE tends to persist over time, it may well express brain parenchymal destruction rather than, for example, temporary oedema [43]. A postmortem study [38] comparing T2-, T1-, and T2*-weighted sequences, FLAIR, and double inversion recovery at 3-T and 7-T showed that none of the five sequences detected significant more lesions than any other sequence and that subpial cortical lesions were far more extensive in histologic inspection than what was detected on 7-T MRI. There is continuous effort in optimisation of imaging techniques. Urushibata et al. [44] recently proposed a magnetisation-prepared two rapid gradient-echo-based sequence, called “fluid and white matter suppression,” which showed better and more uniform WM suppression and more homogeneous GM delineation compared with double inversion recovery sequence, thus representing a potential candidate for better WM and cortical lesion detection. Despite their clinical relevance, focal cortical lesions only represent the tip of the iceberg of cortical pathology in MS. Recent human 7-T studies have demonstrated that a wide range of cortical pathology is present in MS which extends beyond cortical lesions [45, 46]. Mainero et al. [46] have performed a 7-T surface-based analysis of T2* relaxation rates throughout the cortical width, showing that MS patients had significantly increased T2* compared to controls, consistent with cortical myelin and iron loss. These changes were independent from cortical thickness and were mainly confined at 25% depth of the cortical layer and in cortical sulci, providing evidence for the existence of a cortical pathological process driven from the pial surface. Myelin and iron loss is more pronounced within cortical lesions than in normal-appearing cortical GM [47] and in patients with progressive MS compared with relapsing-remitting MS [45]. Although the exact mechanisms underlying GM damage are still unknown, combined 7-T MRI and PET studies [48] have suggested that widespread cortical demyelination is associated with diffuse neuroinflammation within cortical lesions and normal-appearing GM and that the degree of cortical inflammation is associated with worse clinical outcome.

### Leptomeningeal enhancement

**Fig. 2:**
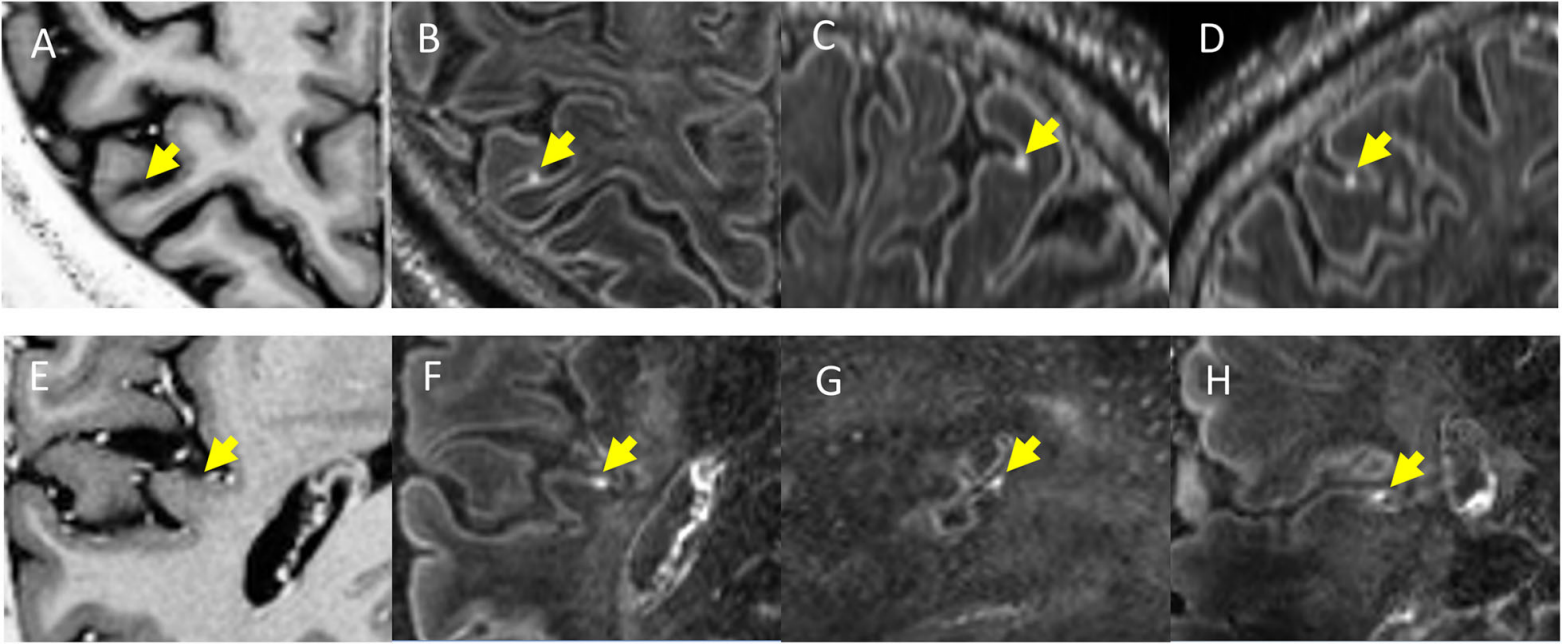
Examples of “nodular” type enhancing foci in multiple sclerosis. In the horizontal rows are displayed two different patients with multiple sclerosis. T1-weighted magnetisation-prepared two rapid gradient echo images (**A**, **E**), along with axial (**D**, **H**), sagittal (**C**, **G**), and coronal (**B**, **F**) slices from contrast-enhanced magnetisation-prepared fluid-attenuated inversion recovery images. The yellow arrows indicate the location of a nodular focus of leptomeningeal contrast enhancement in all three planes and its expected location on magnetisation-prepared two rapid gradient echo images. Published with permission from *J Neurol* [53]

**Fig. 3:**
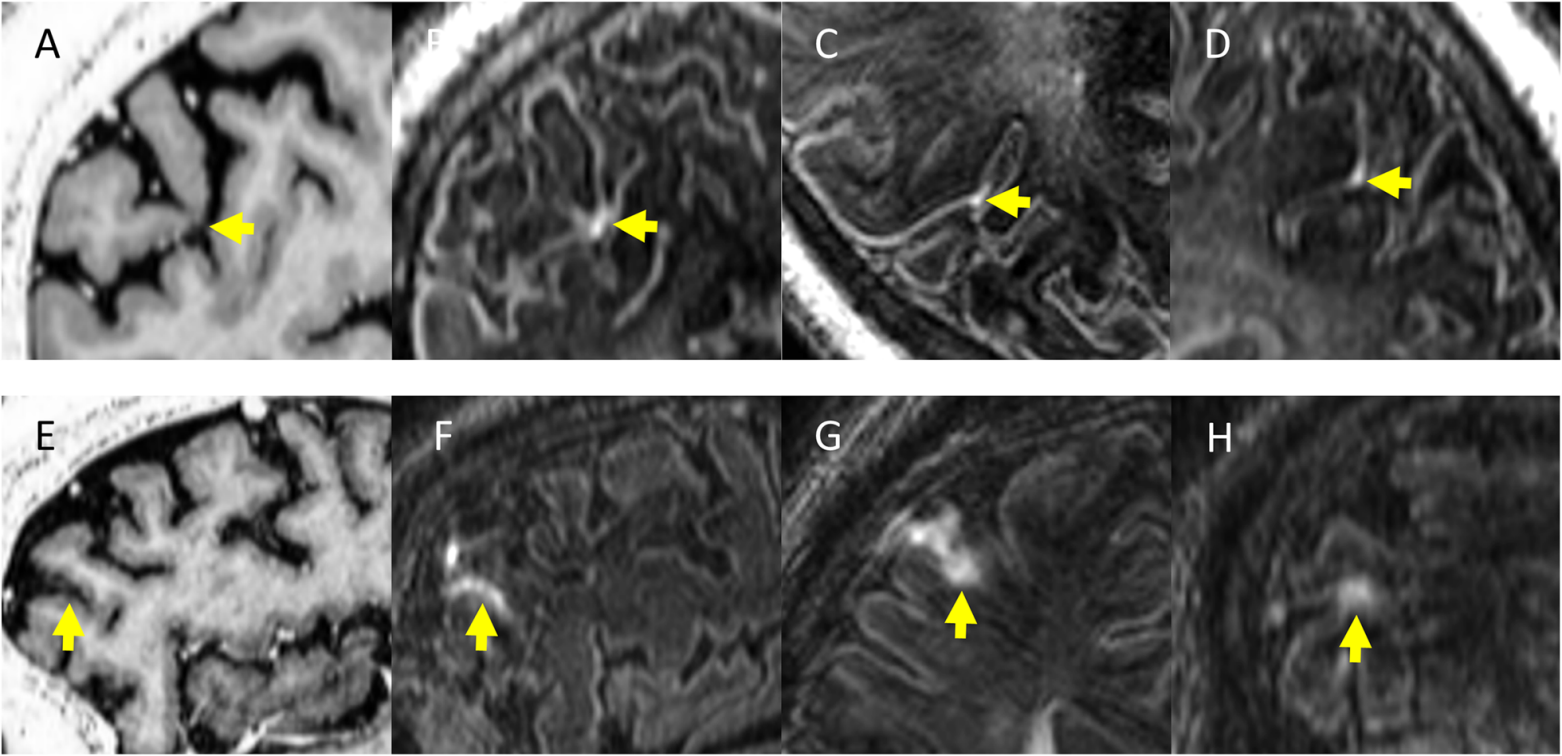
Examples of “spread/fill” type enhancing foci in multiple sclerosis. In the horizontal rows are displayed two different patients. Contrast-enhanced T1-weighted magnetisation-prepared two rapid gradient echo images (**A**, **E**), along with axial (**C**, **G**), sagittal (**D**, **F**), and coronal (**B**, **H**) slices from contrast-enhanced magnetisation-prepared fluid-attenuated inversion recovery images. The yellow arrows indicate the location of a spread/fill focus of leptomeningeal contrast enhancement in all the three planes and its expected location on T1-weighted magnetisation-prepared two rapid gradient echo images. Published with permission from *J Neurol* [53]

A progressively recognised histopathological finding in MS is represented by meningeal inflammation. It is frequently identified in both early and late stages of the disease [49, 50]; it is characterised by aggregates of a variety of immune cells (*i.e.,* T cells, B cells, plasma cells, macrophages, and antigen-presenting cells); and increased numbers of B cells, plasma cells, and follicular dendritic cells are evident in 40% of patients with progressive MS. An important fundamental observation from histopathologic studies is that the meninges are a site of ectopic tertiary lymphoid tissue genesis, organising into B cell follicles. These may be sites of autoantigen presentation and subarachnoid antibody production, which are important for sustaining “smouldering” chronic inflammation in MS [50]. It has to be underlined that meningeal inflammation and its *in vivo* visualisation with MRI is not specific to MS, occurring in other conditions such as infectious and vascular pathology. *In vivo* visualisation with conventional contrast-enhanced T1-weighted images is challenging. Controversial results exist regarding the exact prevalence of meningeal inflammation in MS. Previous attempts on 3-T scanners using contrast-enhanced three-dimensional FLAIR have shown a prevalence varying from 1 to 50% [51, 52]. With three-dimensional FLAIR acquired 10 to 20 min after contrast injection at 7 T, leptomeningeal enhancement (LME) prevalence in MS rises up to 90% [53]. Two principal patterns of LME were identified: “nodular” (round nodules located at the pial surface or subarachnoid space) and “spread/fill” (contrast leakage through the subarachnoid space) (Figs. 2 and 3). Since the latter was associated with cortical GM volume reduction, it may represent blood-meningeal barrier breakdown near sites of meningeal inflammation. It is important to notice that the nodular pattern may be a normal variant, considering that it has been found in a great proportion of healthy controls [53]. Contradictory data exists on the relationship between LME and the presence of cortical lesions in MS. A strong relationship between the presence of cortical lesions and LME was evidenced by some authors [49], though these results were not confirmed in other works [54]. In the absence of a direct comparison between 7-T and 3-T scans in the same subjects, it is not possible to state that 7-T MRI has a higher sensitivity for leptomeningeal enhancement detection. This is a major concern when evaluating LME because for the most part, inflammatory cell clusters in the leptomeningeal compartment are quite small, far below the millimetre-scale spatial resolution of *in vivo* MRI [52]. The wide differences in LME prevalence observed between 3-T and 7-T studies may be also protocol-driven, as previous works had shorter or variable delays between contrast administration and scanning [52]. Delayed scanning is likely necessary, as this provides time for gadolinium to leave the arterial vascular pool, leak through areas of barrier breakdown, and collect in regions of slow CSF flow. Despite the absence of a consensus on the required minimum delay acquisition time, a 20-min interval has been proposed [53]. Another difference among recently published studies is that some works did not acquired unenhanced FLAIR [52]. Without the use of an unenhanced image, it is likely that the true prevalence of LME at 3-T may be less than previously reported. LME detection modality also varies widely: some authors used subtraction techniques [54] whereas others did not [49, 52]. In conclusion, further work is necessary in order to investigate the real prevalence and relevance of LME in MS and to elucidate the ideal MRI protocol to image meningeal inflammation.

### Multinuclear MRI

The application of multinuclear imaging such as sodium (^23^Na) and phosphorus (^31^P) MRI at 7-T is of great advantage due to the low brain concentration of these nuclei. The redistribution of ^23^Na channels from the Ranvier nodes to long segments of demyelinated membrane acts as an adaptive mechanism in order to preserve action potential conduction and facilitate recovery of neurological deficits. However, the consequent huge burden on the axonal metabolism increases the risk of axonal damage secondary to energy deprivation [55]. This is further complicated by mitochondrial dysfunction and adenosine triphosphate deficit, which contributes to axonal accumulation of sodium ions followed by toxic levels of calcium and neurodegeneration. Unfortunately, the ^23^Na signal is 30,000 times lower than that of protons; in addition, in the brain, ^23^Na has a two-compartment distribution with a higher concentration (140 mmol/L) in the extracellular space and a lower concentration (from 10 to 15 mmol/L) in the intracellular space. Therefore, high- and ultra-high-field magnets are very useful to improve sensitivity of *in vivo* ^23^Na MRI, although a direct comparison between the two scanners has not yet been evaluated. It has been shown [56, 57] that patients with MS present with a widespread brain increase of total tissue ^23^Na concentration, possibly reflecting changes in cellular and metabolic integrity of both lesions and normal-appearing brain tissue [58]. Intracellular ^23^Na concentration could be considered as a biomarker of functional tissue changes since it is affected by ^23^Na exchange across cell membrane induced by alterations of tissue metabolism. Single and triple-quantum filtered ^23^Na MRI at 7-T [59] were used to study MS patients and to quantify intracellular ^23^Na concentration and intracellular ^23^Na volume fraction, an indirect measure of extracellular ^23^Na concentration [60]. The intracellular ^23^Na concentration increase detected in the brain WM of patients compared to healthy controls suggested the presence of axonal metabolic dysfunction, thus allowing the speculation that intracellular ^23^Na concentration accumulation might reflect a reversible pathological phenomenon, that could be reversed to a normal condition with appropriate therapeutic interventions. In conclusion, ^23^Na imaging could allow an earlier detection of axonal pathology and might be included in trials assessing the effect neuroprotective drugs. (^31^P) MRI gives specific insight into the energy and membrane metabolism and enables detection of adenosine triphosphate (ATP), free phosphate (Pi), phosphocreatine (PCr), phosphorylethalonamine (PE), glycero-phosphorylethalonamine (GPE), phophorylcholine (PC), glycerophosphorylcholine (GPC), and measuring of pH.

## Other advanced MRI techniques

Diffusion-weighted imaging (DWI) could take many advantages of the use of ultra-high-field magnets. 7-T diffusion tensor imaging (DTI) can benefit from higher spatial resolution and reduction of partial volume effects, thus enabling better separation of fibre bundles in WM and GM and allowing a better characterisation of anatomical connectivity. However, shorter T2 and T2* relaxation times, increased B1+ inhomogeneity, resulting in signal loss in cerebellar and temporal lobe regions, and increased power deposition limit the ability to reduce repetition time, thus increasing the acquisition time [61]. In MS, DTI have the potential to provide more details regarding the microstructural environment of GM, disentangling the relative contribution of inflammatory infiltrates, myelin loss, and axonal degeneration [62]. Resting state functional magnetic imaging is another technique that could benefit from ultra-high-field magnet. Indeed there is a supra-linear increase in the blood oxygenation level-dependent (BOLD) signal change with the static magnetic field strength (B0) increase. Since a large volume coverage is usually required, the limitation at 7-T is represented by the heterogeneity of the radiofrequency (RF) transmission field (Bþ1) which, if not corrected, would result in nonuniform flip angles and tissue contrast across the whole brain. The consequence is a reduced performance in the detection of BOLD signal mostly in the inferior and temporal brain regions [63]. Arterial spin labelling (ASL), which employs magnetically labelled arterial blood water as an endogenous tracer, could take many advantages from 7-T MRI. ASL time-series consist of pairs of label and control images, whose subtraction produces a signal proportional to the local tissue perfusion while their average represents the BOLD signal. Performing ASL at 7-T allows a SNR increase, which improves WM perfusion measurements. Moreover, the increased longitudinal relaxation times at higher fields reduce label decay during postlabelling delay (PLD) and image acquisition leading to higher perfusion SNR. These advantages allow acquiring larger brain coverage and/or using longer PLD to avoid vascular artefacts, which are due to incomplete transfer of the labelled blood from the arterial tree to the local tissue. The advantages of 7-T MRI for BOLD imaging render simultaneous CBF and BOLD imaging using ASL at 7-T particularly attractive [64]. While common MRI methods benefit from increased SNR only, MRI spectroscopy (MRSI) also gains spectral resolution. MRSI benefits include a gain in SNR, a larger frequency dispersion, and a reduction in J-coupling for strongly coupled spin systems. This significantly improves both the number and the accuracy of detectable brain metabolites, thereby making possible new clinical applications [65]. In MS, the assessment of lower glutathione levels in lesions and the GM has become feasible for the first time, using 7 T. Metabolic alterations in glutathione levels and other metabolites occur even beyond T2-visible MS lesions and may thus predict the location of lesion formation [66].

## Spinal cord imaging

**Fig. 4:**
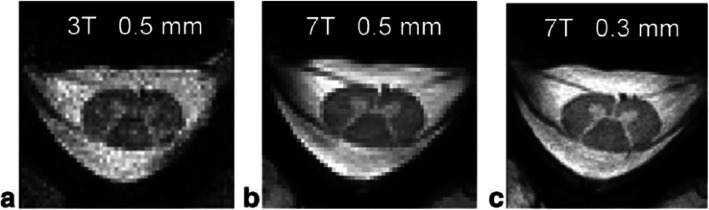
3 T *versus* 7-T MRI of C5-C6 spine in a healthy volunteer: 3-T T2 image with 0.5 × 0.5 × 3 mm^3^ resolution (**a**); 7-T T2 image with 0.5 × 0.5 × 3 mm^3^ resolution (**b**); 7-T T2 image with 0.3 × 0.3 × 3 mm^3^ resolution (**c**). Published with permission of the *International Society of Magnetic Resonance in Medicine* [67]

The spinal cord is affected in nearly 80% of MS patients, and its involvement is responsible for most of clinical disabilities. Considering the small diameter of the spinal cord (about 1 cm), it is self-evident that a higher spatial resolution could minimise partial volume effects between GM and WM. Although MRI of the cord could theoretically benefit from increased signal-to-noise ratio and contrast-to-noise ratio at 7-T (Fig. 4), there are still considerable technical challenges when the spinal cord is evaluated. First, the quality of the MRI data is fundamentally limited by the radiofrequency coils. Second, the effects of small variations in tissue susceptibility are increased at ultra-high-field MRI, leading to greater changes in image contrast. As a consequence, when imaging the spine, the proximity to regions such as the neck, thorax, and lungs is an issue. Third, physiological noise due to respiration, cardiac pulsatility/movement, swallowing, cerebrospinal fluid flow, and bulk movement creates various artefacts. Last, due to the anatomy of the spinal cord, it is difficult to find a balance between the need for a high in-plane resolution, due to the small cross-sectional area, and the elongated shape, which demands a large field of view in the foot-to-head direction. Furthermore, the often curved geometry of the spinal cord makes it difficult to implement the directionally dependent demands on resolution and field of view consistently over all segments [68]. As stated previously, spinal cord imaging at 7-T is challenging and the experience with 7-T spinal cord imaging in MS is limited. A small comparative study of spinal cord imaging of MS patients at 3-T and 7-T has shown that 7-T images provide a better anatomical visualisation of tiny details such as the nerve roots and a better demarcation of GM *versus* WM, and it improves by 50% spinal cord MS lesion detection [69]. 7-T MRI with quantitative measurements has been used to try to differentiate the pathological substrate of MS such as demyelination, remyelination, axonal loss, gliosis, and oedema in the spinal cord [70]. Postmortem spinal cord evaluation of four MS patients showed a strong correlation between myelin content and axonal density with magnetisation transfer, T1-weighed, proton density-weighted, and diffusion anisotropy sequences [70]. Further studies comparing 7-T imaging and 3-T methods for spinal cord lesion detection and segmentation are warranted.

## Lesion characterisation in NMOSD

**Fig. 5:**
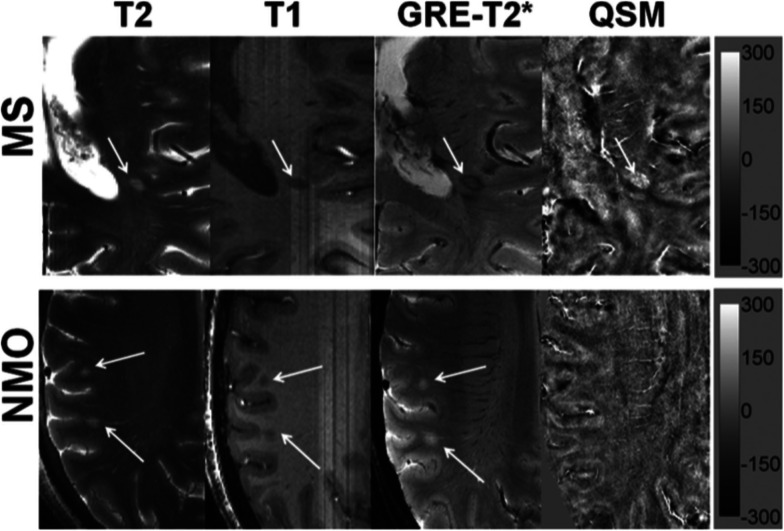
Distinguishing multiple sclerosis (MS) from neuromyelitis optica (NMO) lesions. In the upper row, a patient with multiple sclerosis, from left to right: axial T2-weighted image (T2) with a hyperintense lesion (white arrow) traversed by an ill-defined central venule; the same lesion appears hypointense on a T1-weighted magnetisation-prepared rapid gradient echo image (T1), with hypointense peripheral rim and iso-to-hypointense central core traversed by a well-defined venule on a gradient-echo T2*-weighted image (GRE-T2*), and hyperintense on quantitative susceptibility mapping (QSM). Hypointense signal intensity within the lesion on T2*-weighted image and hyperintensity on QSM suggest iron accumulation. In the lower row, a patient with NMO, from left to right: axial T2-weighted image with two round hyperintense lesions (white arrows); the same lesions appear hypointense on T1, hyperintense on GRE-T2*, and isointense on QSM, thus inconspicuous with iron accumulation. The scale bar is for the QSM image with units of parts per billion. Published with permission from *AJNR Am J Neuroradiol* [74]

WM lesions are reported to be present in up to 68% of NMO patients. Some of these lesions are located in sites with high AQP4 expression such as the periventricular and hypothalamic areas but the vast majority consists in unspecific WM lesions [71]. As already mentioned, early 7-T MRI studies have shown that 60–80% of MS lesions are traversed by a central venule. On the contrary, only a small minority of NMOSD lesions contain central venule [71]. It has been demonstrated that only a minority of NMOSD brain lesions were neighboured (and not centred) by a vein (35%) and hypointense rings surrounding lesions were very rarely detectable (2%) [71]. The use of susceptibility-weighted imaging-filtered phase images has further allowed to characterise lesion morphology both in MS and NMOSD, confirming that in the latter a paramagnetic phase change, in rim-like or nodular fashion, is virtually absent compared to MS (2%) [72]. Interestingly, a reduced periventricular venous density has been demonstrated in MS but not in NMOSD, showing that the reduced proportion of lesion containing a venule is not attributable to a minor venous representation in the latter [73]. The use of quantitative susceptibility mapping has allowed to identify additional morphological differences in iron deposition in the lesions of the two conditions [74] (Fig. 5). In NMOSD lesions, no iron deposition was identified in any lesion, while in MS different subtypes of iron deposition were seen depending on MS plaques stages, thus reflecting a different physiopathology. This finding is not surprising and reflects the notion derived from pathological studies which have showed that NMOSD lesions are characterised by a single phenotype with prominent AQP4 astrocyte loss [75]. Conversely, as already stated, in MS lesions, iron deposition may vary among individual lesions on the basis of their age and inflammatory status (*e.g.*, iron edge deposition in reactive, slowly expanding lesions) [14]. Cortical pathology is another hallmark of MS and its evaluation in NMOSD and has been investigated both on 3-T and 7-T MRI, clearly showing that there is no GM involvement in this pathology spectrum [71]. Discordant opinions exist regarding the integrity of the normal-appearing white matter in NMOSD, with some studies suggesting no occult brain damage in NMO [76] while others showing the presence of widespread normal-appearing WM damage, although to a lesser extent than in MS [77]. In light of the above mentioned works, 7-T MRI might be used in the near future as an useful tool to help in differentiating NMOSD from MS.

## Other neurological disorders: systemic autoimmune diseases

7-T MRI has also been used to investigate both common and rare systemic autoimmune disease that may affect the CNS and can be misdiagnosed as MS. Susac syndrome is a disease characterised by encephalopathy, sensorineural hearing loss, and visual deficits. Its natural course may be self-limiting, monophasic, fluctuating, or relapsing. Typical routine MRI features are the presence of supratentorial hyperintense lesions on T2-weighted images, which mainly involve the central parts of the corpus callosum (“snowballs”) and also the deep grey matter; moreover, parenchymal and leptomeningeal contrast enhancement is frequently encountered [78]. Based on clinical and conventional MRI characteristics, differential diagnosis with MS may be challenging in clinical practice. 7-T MRI has the potential to differentiate these two conditions based on a more accurate lesion morphology. Indeed, lesions in Susac syndrome are not venule-centred and do not present a hypointense ring on T2*-weighted images [72]. Moreover, in Susac syndrome, calossal lesions are hyperintense in the centre and surrounded by a ring-like signal extinction on 7-T T2-weighted images and are more hypointense than MS lesions, reflecting a greater tissue degradation [79]. In systemic lupus erythematosus, nonspecific periventricular or deep WM hyperintensities on T2-weighted images are frequently seen on conventional MRI, making differential diagnosis with MS challenging. In one study, 7-T MRI has allowed to identify cortical-subcortical hyperintensities on T1-weighted images, not visible on conventional MRI, suggestive for microvascular lesions, which are a hallmark of systemic lupus erythematosus pathology, thus allowing to diagnose early neurological involvement in this disease [80]. To date, no studies on acute demyelinating encephalopathy on 7-T MRI are available. Since ADEM may resemble highly active MS onset, ultra-high-field MRI may be a tool for disease characterisation and differential diagnosis.

## Conclusion

7-T MRI has improved the ability to detect smaller and earlier MS lesions, in both WM and GM, and to improve their localisation. Ultra-high-field MRI has provided a new window into MS pathology, allowing a better characterisation of lesion formation, cortical damage, and inflammation within the meninges. The CVS might serve as a novel MRI biomarker to improve the accuracy and speed of MS diagnosis. Metabolic imaging as well as imaging of nuclei other than protons are ready for applications and can provide unique information to elucidate the mechanisms underlying the disease. Continued technical development of new signal transmission and readout methods is needed to overcome the limitations of performing 7-T MRI within comfortable acquisition times and accepted safety limits. Additional clinical studies are needed to demonstrate the value of 7-T for disease diagnosis, prognosis, treatment, and management.

## Data Availability

Not applicable.

## Abbreviations

AQP: Aquaporin
BBB: Blood-brain barrier
CNS: Central nervous system
CSF: Cerebral spinal fluid
CVS: Central vein sign
FLAIR: Fluid-attenuated inversion recovery
GM: Grey matter
LME: Leptomeningeal enhancement
MRI: Magnetic resonance imaging
MS: Multiple sclerosis
NMOSD: Neuromyelitis optica spectrum disorders
PET: Positron emission tomography
WM: White matter
